# Automatic control of finite element models for temperature-controlled radiofrequency ablation

**DOI:** 10.1186/1475-925X-4-42

**Published:** 2005-07-14

**Authors:** Dieter Haemmerich, John G Webster

**Affiliations:** 1 Division of Pediatric Cardiology, Medical University of South Carolina, 165 Ashley Ave., Charleston, SC 29425, USA; 2 Department of Bioengineering, Clemson University, Clemson, SC 29634, USA; 3 Department of Biomedical Engineering, University of Wisconsin, 1550 Engineering Dr., Madison, WI 53706, USA

**Keywords:** ablation, liver ablation, hepatic ablation, radiofrequency ablation, RF ablation, finite element method

## Abstract

**Background:**

The finite element method (FEM) has been used to simulate cardiac and hepatic radiofrequency (RF) ablation. The FEM allows modeling of complex geometries that cannot be solved by analytical methods or finite difference models. In both hepatic and cardiac RF ablation a common control mode is temperature-controlled mode. Commercial FEM packages don't support automating temperature control. Most researchers manually control the applied power by trial and error to keep the tip temperature of the electrodes constant.

**Methods:**

We implemented a PI controller in a control program written in C++. The program checks the tip temperature after each step and controls the applied voltage to keep temperature constant. We created a closed loop system consisting of a FEM model and the software controlling the applied voltage. The control parameters for the controller were optimized using a closed loop system simulation.

**Results:**

We present results of a temperature controlled 3-D FEM model of a RITA model 30 electrode. The control software effectively controlled applied voltage in the FEM model to obtain, and keep electrodes at target temperature of 100°C. The closed loop system simulation output closely correlated with the FEM model, and allowed us to optimize control parameters.

**Discussion:**

The closed loop control of the FEM model allowed us to implement temperature controlled RF ablation with minimal user input.

## Background

**Figure 1:**
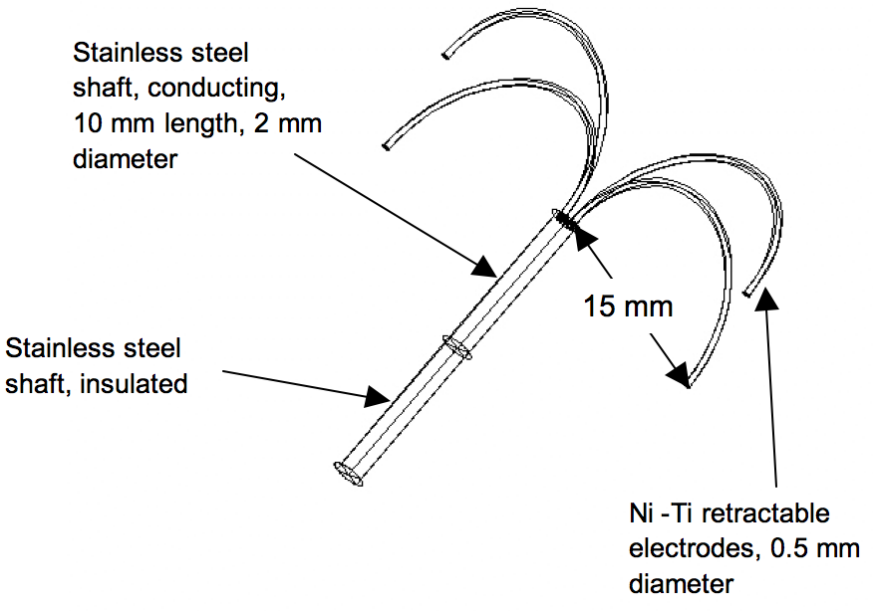
Geometry of fully deployed Rita model 30 umbrella probe used in FEM model. The prongs and the distal 10 mm of the shaft conduct RF current.

Radio-frequency (RF) ablation has become of considerable interest as a minimally invasive treatment for primary and metastatic liver tumors. Hepatocellular carcinoma is one of the most common malignancies, worldwide with an estimated annual number of 500,000 deaths [1]. Surgical resection offers the best chance of long-term survival, but is rarely possible. In many patients with cirrhosis or with multiple tumors, hepatic reserve is inadequate to tolerate resection and alternative means of treatment are necessary [2]. In RF ablation, RF current of 450 to 500 kHz is delivered to the tissue via electrodes inserted percutaneously or during surgery. Different modes of controlling the electromagnetic power delivered to tissue can be utilized. Power-controlled mode (*P *= constant), temperature-controlled mode (*T *= constant) and impedance-controlled mode (*Z *< constant) are commonly used. The electromagnetic energy is converted to heat by resistive heating. Tissue damage can occur at temperatures above 43°C with heating times of several hours; at 50°C cell necrosis occurs after ~3 min [12]. A commonly used mode is temperature-controlled ablation, where the tip temperature of the electrodes is kept at a predetermined value, usually around 100°C. The hepatic electrodes (Fig. 1) used for temperature-controlled ablation have temperature sensing elements (thermistors or thermocouples) embedded in the prong tips. The sensors report temperature back to the generator, which then applies an appropriate amount of power to the electrode to keep the temperature constant.

Researchers have been using the finite element method (FEM) to simulate both cardiac and hepatic RF ablation [3-11]. When using the FEM to model temperature-controlled ablation, the applied voltage has to be adjusted to keep the tip temperature constant. Previously, most researchers used manual adjustment of applied voltage and trial-and-error methods to perform temperature-controlled ablation [3,5-8]. Implementation of temperature-controlled feedback in models is of importance to obtain results comparable to clinical devices that use this type of control. We modeled a clinically used hepatic ablation electrode (15 gauge, RITA medical systems model 30) as described previously [6]. We implemented a control algorithm for a PI controller – a commonly used controller type – in a C++ program to change the applied voltage between the time steps. We used a closed loop system simulation to optimize control parameters for the PI controller.

## Methods

### 1. Finite element method

RF ablation destroys tissue by thermal energy, which is converted from electric energy. The current flows from the conductive electrode through the tissue to a surface dispersive electrode. Tissue in close vicinity of the electrode tip is heated by resistive heating.

The heating of tissue during RF ablation is governed by the bioheat equation:



where *ρ *is the density (kg/m^3^), *c *is the specific heat (J/(kg·K)), and *k *is the thermal conductivity (W/(m·K)). ***J ***is the current density (A/m^2^) and ***E ***is the electric field intensity (V/m). *T*_bl _is the temperature of blood, *ρ*_bl _is the blood density (kg/m^3^), *c*_bl _is the specific heat of the blood (J/(kg·K)), and *w*_bl _is the blood perfusion (1/s). *h*_bl _is the convective heat transfer coefficient accounting for the blood perfusion. *Q*_m _(W/m^3^) is the energy generated by metabolic processes and was neglected since it is small compared to the other terms [13]. Equation (1) defines the solution in the spatial domain encompassing electrodes and tissue.

Tissue properties were assumed temperature independent and are described in more detail in [6]. We used the commercial software ABAQUS/Standard 6.3 (Hibbitt, Karlsson & Sorensen, Inc., Pawtucket, RI) for solving the coupled thermo-electrical analysis. All analysis was performed on a SUN Blade-1000 workstation equipped with 2.5 GB of RAM and 80 GB of hard disk space.

Fig. 1 shows the geometry of the RITA model 30, 4-prong electrode we used in our models. The electrode was placed within a cylinder (80 mm diameter, 50 mm length) of liver tissue. The outer surfaces of the cylinder were set to 37°C (thermal boundary condition), and 0 V (electrical boundary condition). We performed quasi-static analysis. Due to the symmetry of the arrangement, we could reduce computing time by only modeling a quarter of the cylinder. We used the same model as in a previous study [3]; the model consisted of ~35,000 tetrahedral elements and ~7,000 nodes. The node spacing was small next to the electrode (0.2 mm) and larger at the model boundary (2 mm). Perfusion was included in the model according to the Pennes model [14]. The blood perfusion *w*bl used in this model was 6.4·10^-3 ^1/s [15].

An input file was submitted to ABAQUS, in which geometry, material properties, step time and boundary conditions were specified. The applied voltage was one of the boundary conditions and remained constant during each step. ABAQUS created a results file in which temperatures of all nodes of the FEM model at the end of the step were written. We created a C++ program that reads the temperature at the electrode tip from the results file and creates a new input file where the applied voltage is set according to a control algorithm; the C++ program then calls ABAQUS and waits until the next step is completed. We implemented a PI controller in our control algorithm.

### 2. Closed loop system simulation

**Figure 2:**
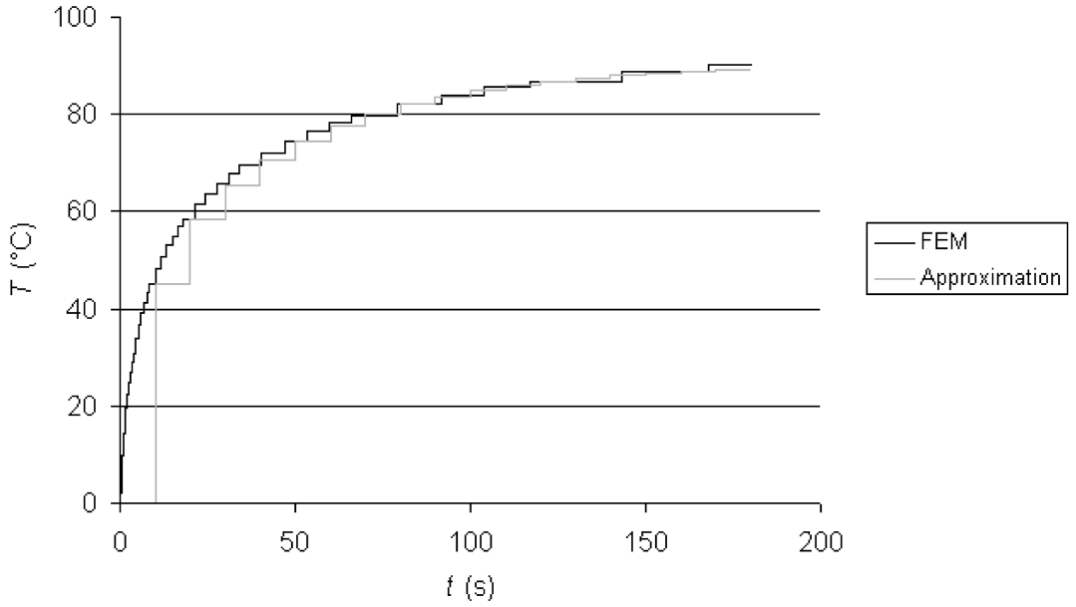
Step response of the original dynamic system (FEM model) and of the approximation.

Since the FEM model takes several hours to complete, using the FEM model to optimize control parameters was not feasible. Therefore we used a closed loop simulation to adjust parameters of the controller, and then used these parameters in the FEM model. Before implementing the controller, we analyzed the dynamic system (i.e. the FEM model) to be controlled. This system consisted of the ablation electrode, tissue, and dispersive electrode. The input variable of the system was the voltage applied to the electrode. The output variable was the temperature measured at the tip of the electrode. Initially, we determined the step response by applying a constant voltage of 25 V for 180 s. We then approximated the transfer function of this system by a time-discrete transfer function of the following form:



We used the control system simulation software ANA 2.52 (Freeware, Dept. of Control Engineering, Tech. Univ. Vienna/Austria) to analyze the control system. This software allowed us to approximate the system from its step response by a recursive least square algorithm and gave the parameters *a*_0_, *a*_1_, *a*_2_, *b*_1 _and *b*_2 _of (2). Fig. 2 shows the step responses of the original dynamic system (i.e. the FEM model) and of the approximation according to (2). The parameters used for the approximation in (2) were: *a*_0 _= 1.0, *a*_1 _= -0.959, *a*_2 _= 0.127, *b*_1 _= 1.734, *b*_2 _= -1.150. The sampling time of the approximated system was 10 s, since that was also the sampling time used later in the digital PI controller. This ensured a more accurate simulation model of the closed-loop control system.

**Figure 3:**
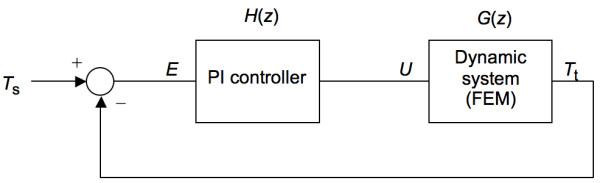
Closed loop system incorporating a PI controller and the dynamic system (FEM model).

Once we had identified an approximation of the dynamic system (FEM model), we designed a feedback control system. There are different ways of controlling the tip temperature such as PID control, adaptive control, neural network prediction control and fuzzy logic control. We chose the relatively simple PI controller for our control system. Fig. 3 shows the complete closed-loop control system. *T*_s _is the desired set tip temperature and *T*_t _is the current tip temperature. The input of the PI controller *e *= *T*_s _- *T*_t_. The output of the PI controller *u *(corresponds to the applied voltage) is fed into the dynamic system.

The transfer function of the PI controller is:



Equation (3) can be described in the time-domain by:



To implement this controller in software, we have to use the discrete time-domain version of (4),



The second term of (5) represents the approximation of the integral term in (4) by trapezoidal numerical integration. The behavior of the PI controller is determined by the two parameters *K*_p _and *K*_i_.

We simulated the behavior of the closed-loop system with the software ANA. From in-vivo experiments in pigs with the RITA 500 generator and the Model 30 electrode we determined that it takes between 1 and 2 min for the tip temperature to reach a target temperature of 100°C, which is the temperature used in clinical cases at our institution. The RITA 500 generator does not allow recording of the tip temperature signal, so we don't have exact data of the tip temperature over time. We empirically chose parameters for the PI controller to minimize overshoot and obtain similar temporal behavior as in the in-vivo experiments.

The parameters we used for the PI controller were *K*_p _= 0.02, *K*_i _= 0.0064.

**Figure 4:**
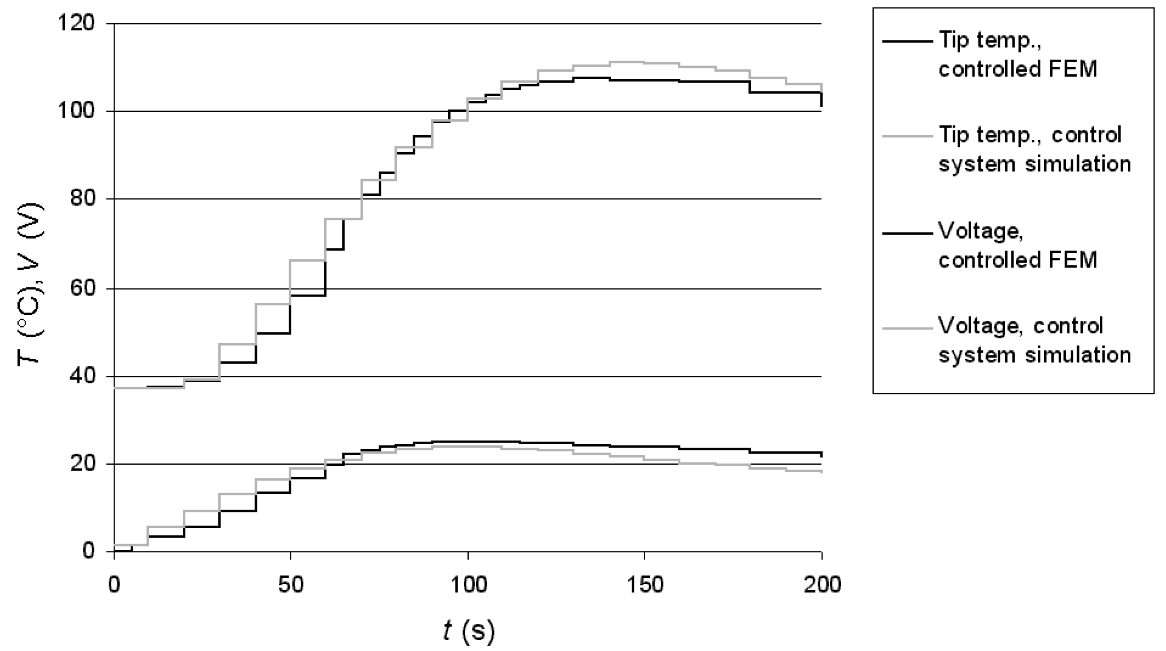
Temporal behavior of tip temperature and applied voltage of the closed loop system. The upper curves show the tip temperature, the lower curves show the applied voltage. The light curves show the results of the control system simulation. The dark curves show the results of the FEM model controlled by the control software.

Fig. 4 shows the results of the closed-loop control system simulation. The target tip temperature of 100°C was reached 100 s after start of the ablation. The maximum overshoot was 11%, which was reached after 148 s. The maximum voltage of 24.5 V was applied after 100 s.

### 3. Controlled FEM model

We implemented the PI controller with the parameters resulting from the control system simulation in a control program written in C++. The software control program determined the applied voltage and the step time. An input file for ABAQUS was created and ABAQUS solved the FEM model. ABAQUS created a result file, which included the temperatures at all nodes.

The tip temperature was read from the result file by the control program. The program then determined the applied voltage and step size for the next step, and ABAQUS was restarted with the modified input file. With decreasing step size (i.e. time that ABAQUS simulated the model with constant voltage) total computation time gets longer. As a compromise, we chose 10 s as the initial step size. Note that the FEM solver divided each step into smaller increments, starting at 0.05s. Also, the FEM solver performed convergence tests to ensure that the increment size was sufficiently small.

**Figure 6:**
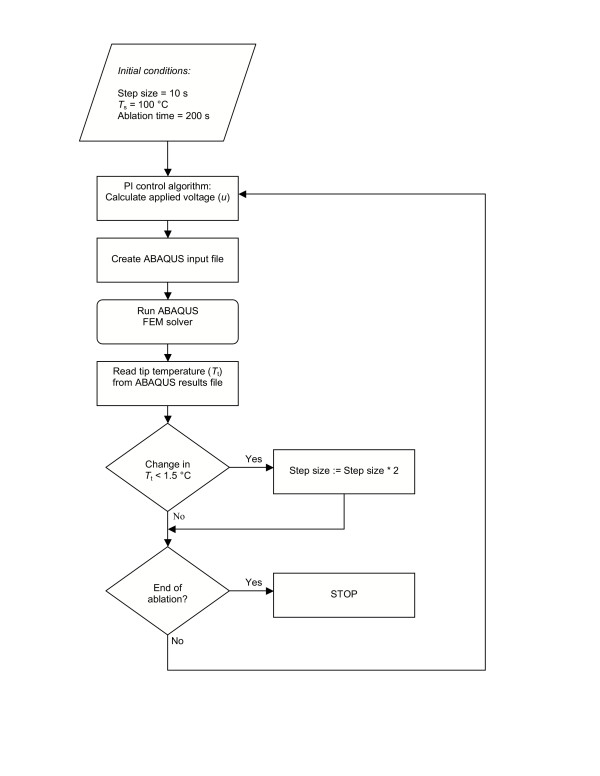
Flowchart of the control software implementing a PI control algorithm.

This scheme was repeated until the ablation had been simulated for the desired time. Fig. 6 is a flow chart of the algorithm implemented in the control program. The initial step size was 10 s. Subsequently, the step size increased once the tip temperature change between the steps decreased below a certain value.

We simulated ablation for 12 min using the FEM model and the control program. The simulation took 250 min to run and was divided into 35 steps. The individual steps took between 6 and 11 min to run in ABAQUS. Without using the control program, manual interaction would be necessary after each step to manually change the input file for ABAQUS and apply a new input voltage and set the step size. In this case the operator would have to check the results file after each step (i.e. in 6 to 11 min intervals), modify the input file and restart ABAQUS.

## Results and Discussion

**Figure 5:**
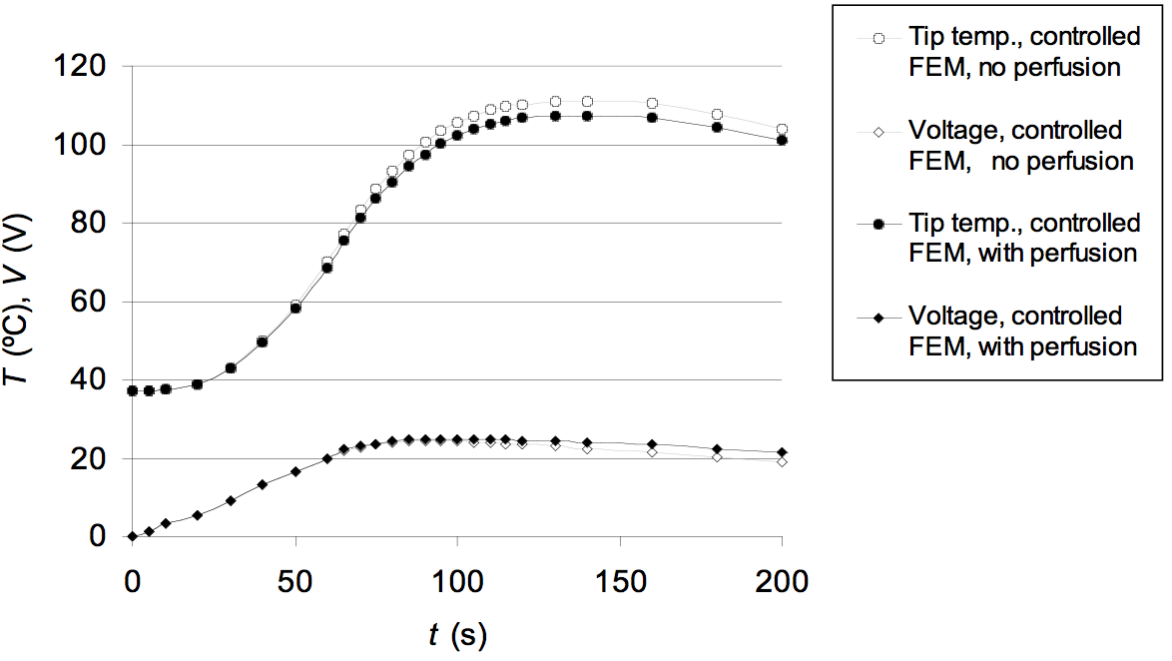
Temporal behavior of tip temperature and applied voltage of the closed loop system. The upper curves show the tip temperature, the lower curves show the applied voltage. The light curves show the results of the controlled FEM model without perfusion. The dark curves show the results of the controlled FEM model incorporating perfusion.

Fig. 4 shows the resulting tip temperature and the applied voltage for the first 200 s of the closed loop system consisting of the FEM model and the control software. No results are shown after 200 s since there was little change once the set tip temperature was reached. Fig. 4 also shows the results of the control system simulation. There is good correlation between the control simulation and the closed loop system consisting of the FEM model and the control software. We explain deviations between the two by inaccuracies of the approximation (see (2)) and differences in step sizes. In the control system simulation a constant sampling time (i.e. step time) of 10 s was used. However, the algorithm implemented in the control program did not use constant step times. Step time was increased if the change in tip temperature between the steps was below a certain value. Note that the parameters of the controller must be modified when boundary conditions (e.g. perfusion), electrode geometry etc. are changed. Otherwise there will be changes in the dynamic behavior of the closed-loop system. Fig. 5 shows the temperature and voltage of the FEM model controlled by the same PI controller for two cases. The light graphs show the behavior without incorporating perfusion in the FEM model, the dark graphs show the behavior of the same FEM model with perfusion (as in Fig. 4). Both the overshoot and the settling time of tip temperature for the case with perfusion were higher. Also, the voltage required to keep the tip temperature at the set temperature value was higher because the perfusion carried heat away. To obtain the same performance during the initial period in both cases, different controllers had to be used, e.g. the parameters of the PI controller had to be modified. Our model only included a quarter of the actual electrode. In models where non-uniform heating due to vessels occurs, the electrodes will obtain different temperatures [6]. In this case, the temperature of the hottest electrode should be used for control to avoid tissue overheating.

In hepatic RF ablation, ablation times clinically used go up to 35 min. Since the heat-up period is comparably much smaller (1 to 2 min.), it is not of essential importance that the temporal behavior of the control algorithm reproduces the control algorithm used in clinical devices during the heat-up period. From our experience, the temperature distribution in the FEM model reaches close to steady state at the end of the simulation due to the long simulation times. As long as the tip temperature is kept within a small range around the target temperature after the initial heat-up period, the model results (i.e. final temperature distribution) should not differ significantly. However, with knowledge of the actual control parameters and algorithms of commercial devices (e.g. obtained from measurements), accurate simulations of these devices is possible using our methods.

## Conclusion

We implemented a closed loop control system to a FEM model, to automate simulation of temperature-controlled RF ablation. We further used a closed loop control system simulation to optimize control parameters. Previously, researchers often applied constant power, or used time-consuming trial-and-error methods to determine required voltage. Furthermore, if control parameters and algorithms of commercial devices are known or can be measured, an accurate simulation of commercial devices is possible.

## Authors' contributions

DH carried out computer simulations. JG participated in design of the study. All authors read and approved the final manuscript.
